# Bone Mineral Density in Different Menopause Stages is Associated with Follicle Stimulating Hormone Levels in Healthy Women

**DOI:** 10.3390/ijerph18031200

**Published:** 2021-01-29

**Authors:** Young-Min Park, Catherine M. Jankowski, Christine M. Swanson, Kerry L. Hildreth, Wendy M. Kohrt, Kerrie L. Moreau

**Affiliations:** 1 Department of Medicine, Division of Geriatric Medicine, University of Colorado Anschutz Medical Campus, Aurora, CO 80045, USA; ypark@inu.ac.kr (Y.-M.P.); catherine.jankowski@cuanschutz.edu (C.M.J.); kerry.hildreth@cuanschutz.edu (K.L.H.); wendy.kohrt@cuanschutz.edu (W.M.K.); 2 Division of Health and Kinesiology, Incheon National University, Incheon 22012, Korea; 3 College of Nursing, University of Colorado Anschutz Medical Campus, Aurora, CO 80045, USA; 4 Department of Medicine, Division of Endocrinology, Metabolism and Diabetes, University of Colorado Anschutz Medical Campus, Aurora, CO 80045, USA; christine.swanson@cuanschutz.edu; 5 VA Eastern Colorado Health Care System, Geriatric Research Education and Clinical Center (GRECC), Denver, CO 80045, USA

**Keywords:** perimenopause, bone mineral density, estrogen, follicle stimulating hormone

## Abstract

Although estradiol (E_2_) has been believed to be the most critical factor in the menopause-associated decrease in bone mineral density (BMD), the role of increasing follicle stimulating hormone (FSH) during menopause is relatively unclear. We determined the extent to which hip and lumbar spine BMD differ among the stages of menopause in healthy women, and whether BMD is associated with FSH and E_2_ levels. A cross-sectional study of 141 healthy women classified as premenopausal (Pre; 38 ± 6 yrs; mean ± SD, *n* = 30), early perimenopausal (EPeri; 50 ± 3yrs, *n* = 31), late perimenopausal (LPeri; 50 ± 4yrs, *n* = 30), early postmenopausal (EPost; 55 ± 3yrs, *n* = 24), or late postmenopausal (LPost; 62 ± 4 yrs, *n* = 26), was conducted. Spine/hip BMD and sex hormones were measured using dual-energy X-ray absorptiometry and enzymatic/colorimetric methods, respectively. Compared to EPeri, spine BMD was lower (*p* < 0.05) in LPeri, EPost, and LPost and hip BMD was lower (*p* < 0.05) in EPost and LPost. BMD was inversely associated with FSH (spine: r = −0.341; hip: r = −0.271, *p* < 0.05) and directly associated with E_2_ (spine: r = 0.274; hip: r = 0.256, *p* < 0.05). The menopause-related loss of spine and hip BMD is associated not only with low E_2_ but also higher FSH. Future studies are essential to delineating the mechanisms by which FSH regulates bone health in aging women.

## 1. Introduction

Fractures and the consequent loss of independent living are critical health issues in aging women [1]. The menopause transition, also called perimenopause, is associated with an accelerated loss of bone mineral density (BMD), which increases the risk of osteoporosis and bone fractures [2]. BMD loss starts before the final menstrual period [3], and continues throughout the menopausal transition [4,5]. This fast decline in BMD can be associated with irreversible disruption of bone microarchitecture [6,7] and a greater risk of spine and hip fractures [8]. The goal of the present study was to determine how menopausal stages and levels of sex hormones are associated with BMD at the total hip and lumbar spine.

For many years, declining estradiol (E_2_) was believed to be the most critical direct hormonal regulator of the menopause-associated decline in BMD [9,10,11,12]. However, as E_2_ declines, the negative feedback on follicle stimulating hormone (FSH) released from the anterior pituitary is vanquished, resulting in elevated FSH levels [13]. Mounting pre-clinical [14,15,16] and clinical [3,17,18,19] evidence suggests that the menopause-related increase in FSH is a significant and independent regulator of bone loss. In the “Study of Women’s Health Across the Nation” (SWAN) study [17], FSH was a better predictor of imminent bone loss than E_2_ during the menopause transition. In a cross-sectional study of pre-, peri-, and postmenopausal women, Wu et al. [18] showed that the estimated rate of decrease in lumbar spine BMD within the stages of menopause was related to FSH rather than E_2_, even after adjustment for age and body mass index (BMI). Because of the rapid loss of bone during perimenopause, we used retrospective data from healthy women in various menopausal stages from a previous study [20] to evaluate the associations of FSH and E_2_ with lumbar spine and hip BMD. We hypothesized that the rapid BMD loss during menopause would be more strongly associated with increased FSH than with decreased E_2_. Our study was also to provide a comprehensive evaluation of sex steroid levels in women across the menopause transition with physical activity assessments.

## 2. Materials and Methods

### 2.1. Participants

One hundred and forty-one healthy women aged 30–70 years were included in this study [20]. Menopausal status was assessed by self-reported menstrual cycle history, and categorized according to the Stages of Reproductive Aging Workshop (STRAW) criteria [21], as premenopausal (Pre; *n* = 30; 21–35 d regular menstrual cycles), early perimenopausal (EPeri; *n* = 31; >2 cycles with cycle length changes of ≥7 d), late perimenopausal (LPeri; *n* = 30; ≥2 and <12 months of amenorrhea), early postmenopausal (EPost; *n* = 24; ≤5 yr since menopause), or late postmenopausal (LPost; *n* = 26; >5 yr since menopause). Women included in the study were non-smokers, sedentary, or recreationally active (<3 days/week vigorous exercise), had no history of oral contraceptives or hormone therapy for the previous 6 months, were normotensive, and were healthy as determined by standard blood chemistries, physical examination, medical history, and electrocardiography. Women with a history of or active cancer, estrogen-dependent neoplasms, cardiovascular disease, hysterectomy/oophorectomy, venous thromboembolism, acute liver, and gallbladder disease were excluded. The protocol was approved by the Colorado Multiple Institutional Review Board (COMIRB), and participants provided written informed consent.

### 2.2. Body Composition

Total body lean mass, total body fat mass, total body bone mineral content, and bone mineral density (BMD) of the lumbar spine (L1-4) and hip (femoral neck, greater trochanter, and total) were measured by dual X-ray absorptiometry (DXA, Hologic Discovery, software version 11.2, Hologic Inc, Waltham, MA, USA) as previously described [22]. BMD T-scores were determined using the manufacturer’s internal comparative database, and women were classified as having normal bone density, low bone mass (LBM; T-score < −1.0 and > −2.5), or osteoporosis (T-score ≤ −2.5) at the lumbar spine or hip [23].

### 2.3. Sex Hormones and Inflammatory Marker

All blood was collected following an overnight fast. The lab personnel were blinded and all hormones and inflammatory marker were assessed in duplicate. Serum levels of E_2_, FSH, and progesterone were assessed using chemiluminescense (Beckman Coulter, Inc., Brea, CA, USA). Testosterone and estrone were assessed using 1-step competitive assay and radioimmunoassay, respectively (Beckman Coulter, Inc., Brea, CA, USA). Interleukin-6 (IL6) was measured using colorimetric methods (R&D systems, Inc., Minneapolis, MN, USA). All assays were performed by the Colorado Clinical and Translational Sciences Institute (CCTSI) Clinical and Translational Research Center (CTRC) Core laboratory, which is CAP- and CLIA-accredited. The intra- and inter-assay coefficients of variation have been described previously [24]. The coefficient of variation (CV, 95% CI) for each hormone are as follows: intra-assay CV: estradiol, 4.3%; estrone, 11.5%; FSH, 1.8%; progesterone, 4.4%; testosterone, 2.1%; IL6, 7.8%; and inter-assay CV: estradiol, 8.2%; estrone, 19.8%; FSH, 3.8%; progesterone, 7.9%; testosterone, 5.1%; IL6, 11%. The sensitivity for each hormone is as follows: estradiol, 10 pg/mL; estrone, 10 ng/dL; FSH, 11 uIU/mL; progesterone, 10 ng/dL; testosterone, 17 ng/dL; and IL6, 0.156 pg/mL [24].

### 2.4. Physical Activity, Energy Intake, and Vascular Function

Physical activity level was determined by leisure time physical activity (LTPA) using the Modifiable Activity Questionnaire [25]. The questionnaire included the number of hours spent in sedentary activities and the frequency of participation in different physical activities, and finally calculated these in total metabolic equivalent tasks (MET). Energy intake was determined by 3-day food intake records [26]. The CCTSI Nutrition Core analyzed the dietary food records. Vascular function was determined by brachial artery flow-mediated dilation (FMD) as described previously [27]. FMD was performed according to current guidelines of human FMD assessment [28].

### 2.5. Statistical Analysis

Results are presented as mean ± standard deviation (SD) for normally distributed variables. If variables were skewed (i.e., E_2_, estrone, progesterone, LTPA, and testosterone), median and interquartile ranges were used. The skewed variables were log-transformed for statistical comparison. One-way analysis of variance (ANOVA) was used to determine the main effects of menopause stage on BMD and participant characteristics. For spine and hip BMD outcomes only, the significant main effects were further analyzed by Tukey HSD *post hoc* tests to determine differences among menopause stages. Exploratory analyses were performed using Bivariate Pearson’s correlations to assess the association between spine and hip BMD and sex hormones. Age, sex hormones, energy intake, and physical activity were tested as potential covariates. *p* < 0.05 was considered statistically significant. All data were analyzed using IBM SPSS Statistics version 25.0 (IBM/SPSS, Armonk, NY, USA).

## 3. Results

### 3.1. Population

Significant differences in age, total body lean mass, total body bone mineral content, E_2_, estrone, FSH, brachial FMD, and progesterone concentrations were found among the menopausal stages (Table 1, all *p* < 0.05). There were no significant differences in body weight, height, BMI, total body fat mass, testosterone, energy intake, IL6, and LTPA among the menopausal stages (Table 1).

### 3.2. Spine and Hip BMD Analysis

**Figure 1:**
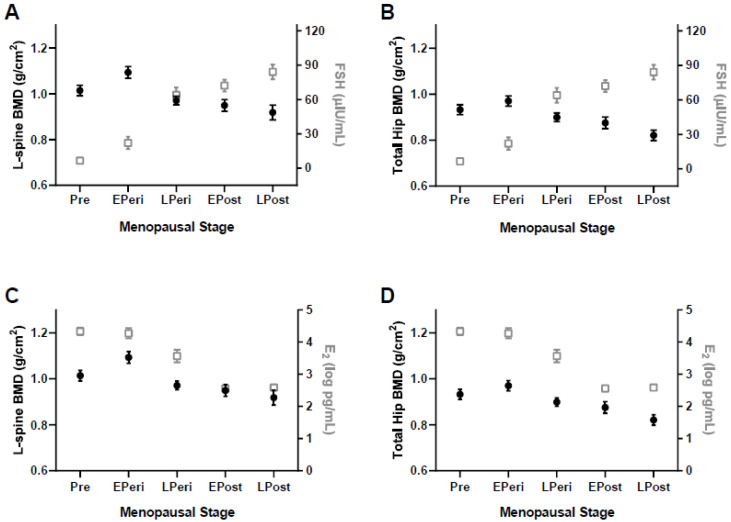
Bone mineral density (BMD) and sex hormones in different menopausal stages. (**A**) Lumbar-spine (L-spine) BMD versus follicle stimulating hormone (FSH), (**B**) total hip BMD versus FSH, (**C**) lumbar-spine BMD versus estradiol (E_2_), and (**D**) total hip BMD versus E_2_. Values are means ± SE. Solid symbols indicate BMD, open symbols indicate hormone levels. Pre = premenopausal; EPeri = early perimenopausal; LPeri = late perimenopausal; EPost = early postmenopausal; LPost = late postmenopausal.

L1-4 BMD and T-score were not significantly different in Pre and EPeri, whereas they were significantly lower in LPeri, EPost, and LPost compared to EPeri (Table 2, all *p* < 0.05). Femoral neck BMD was not significantly different in Pre, EPeri, and LPeri, but was lower in EPost compared to EPeri, and lower in LPost compared to Pre, EPeri, and LPeri (Table 2; all *p* < 0.05). Trochanter and total hip BMD and total hip T-scores were not significantly different in Pre, EPeri, and LPeri, but were lower in EPost compared to EPeri, and lower in LPost compared to Pre and EPeri (Table 2; Figure 1; all *p* < 0.05).

### 3.3. Prevalence of LBM and Osteoporosis

Low bone mass was found in every menopausal stage, with the highest prevalence (54.2%) in EPost. No women in the Pre and EPeri stages had osteoporosis, but nearly 27% of women in LPost had osteoporosis (Table 2). In postmenopausal women, 31% of EPost women were prior hormonal replacement therapy (HRT) users (average duration, 2.9 ± 2.8 yrs), and 48% of LPost women were prior HRT users (average duration, 4.8 ± 3.6 yrs). There were no significant differences in spine L1-4, femoral neck, trochanter, and total hip BMD between non-HRT and HRT users.

### 3.4. Associations

**Figure 2:**
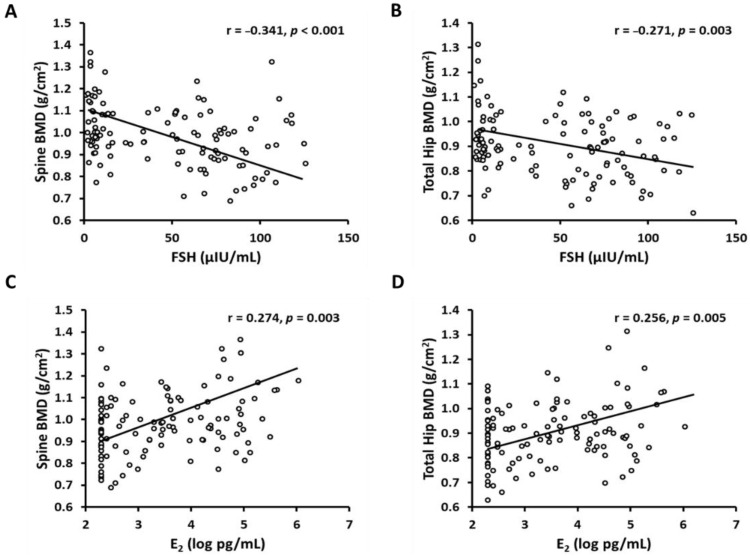
Associations of bone mineral density (BMD) and sex hormones. (**A**) Lumbar-spine BMD versus follicle stimulating hormone (FSH), (**B**) total hip BMD versus FSH, (**C**) lumbar-spine BMD versus estradiol (E_2_), and (**D**) total hip BMD versus E_2_.

Spine and total hip BMD were inversely correlated with FSH (spine: r = −0.341, *p* < 0.001; and hip: r = −0.271, *p* = 0.003; Figure 2) and age (spine: r = −0.147, *p* = 0.084; and hip: r = −0.242, *p* = 0.004; Table 3), but directly correlated with E_2_ (spine: r = 0.274, *p* = 0.003; and hip: r = 0.256, *p* = 0.005; Figure 2) and estrone (spine: r = 0.239, *p* = 0.01; and hip: r = 0.227, *p* = 0.014; Table 3). There were no significant associations between spine or total hip BMD and other variables of interest (i.e., progesterone, testosterone, energy intake, and LTPA; Table 3). The inverse associations of spine and total hip BMD with FSH remained significant or tended to be significant even after adjusting for age, E_2_, estrone, progesterone, testosterone, energy intake, and LTPA (Table 3). The direct correlations of spine and hip BMD with E_2_ remained significant or tended to be significant after adjusting for age, progesterone, testosterone, energy intake, and LTPA, whereas the significant correlations of spine and hip BMD with E_2_ became non-significant after adjusting for FSH and estrone (Table 3).

## 4. Discussion

The present study provides additional cross-sectional evidence that pituitary hormone FSH may play a role in menopause-related bone loss. We corroborate previous data demonstrating that menopause appears to be a vulnerable period for bone loss, even in healthy women. Lower spine BMD appeared in earlier stages of menopause (late perimenopause) than hip BMD (early postmenopause). Finally, lower BMD at both the spine and total hip were significantly, weakly correlated with FSH levels during menopause, and these associations were independent of E_2_.

### 4.1. Menopause, BMD, and Physical Activity

Our results are consistent with prior literature demonstrating BMD decline during menopause. Longitudinal studies [5,29,30], including the SWAN study [5], also reported that spine and hip BMD begin to decline during perimenopause. This multi-site, multi-ethnic study [5] included 1902 women who were pre- or early perimenopausal at baseline, and assessed spine and hip BMD across six annual visits. Little change in BMD occurred during the pre- or early perimenopause. Then, bone loss accelerated during late perimenopause and continued during postmenopause. Similarly, the present study found lower spine BMD in late compared to early perimenopause. Another study [4] of 3302 women (age: 42–52 yrs) also supports our findings in that a rapid, significant loss of spine and hip BMD began one year before the final menstrual period (i.e., perimenopause) and decelerated two years after the final menstrual period. The present study adds to the literature that lower BMD is found in late compared to early perimenopausal women who are otherwise healthy.

Compared to premenopausal, postmenopausal women exhibit a greater prevalence of physical inactivity [31]. Although human [32] and rodent [33] studies demonstrated a menopause-associated decline in physical activity, the present study showed no significant difference in self-reported leisure time physical activity between menopausal stages with great variations of each group. Furthermore, physical activity was not correlated with BMD nor did it influence the associations of FSH or estradiol with BMD. Physical inactivity can exacerbate BMD decline and increase fracture risk. Whether menopause contributes to declines in physical activity after menopause needs to be studied in a larger-cohort study using a more accurate measure of physical activity (e.g., accelerometry).

### 4.2. FSH and BMD

Estradiol levels decline across the menopausal transition, while FSH levels increase [13]. Temporally, the increase in FSH occurs earlier in the menopause transition than the decrease in E_2_. The profound declines in BMD, corresponding with high bone resorption, occur two to three years prior to the final menstrual period, during which FSH levels increase while estrogen levels remain relatively stable [19,34]. Similar to this finding, the present study showed that the greatest differences in FSH (four-fold increase) in the menopause transition occurred between EPeri and LPeri, while E_2_ was stable. In a prior study, women with serum FSH levels greater than 30 IU/L had greater bone turnover markers (suggesting rapid bone loss) compared to age-matched women with lower FSH [35]. Women with hypergonadotropic hypogonadism (mean FSH levels of ~35 IU/L) had greater bone loss compared to those with hypogonadotropic hypogonadism (mean FSH levels of ~8 IU/L) [36]. This potential “FSH threshold” of 30 IU was reached in the LPeri group in the present study, and correlated with the onset of BMD decline at the L-spine and total hip.

Estradiol has been believed to be the most important factor in menopausal BMD loss. However, for the last decade, increasing evidence has suggested the potential role of FSH itself in bone health. Sun et al. [14] identified direct actions of FSH on bone cells to increase osteoclast precursors and osteoclastogenesis, which sensitized the mitogen-activated protein kinase (MAPK), nuclear factor kappa B (NFκB), and akt pathways. Concurrent with this proresorptive effect of FSH, bone loss in ovariectomized rats was augmented by the exogenous treatment of FSH, and attenuated by the administration of FSH antagonist [37,38]. This finding was also supported by the finding that an FSH antibody targeting receptor-binding sites of the FSHβ subunit prevented ovariectomy-induced bone loss in mice [15] by suppressing osteoclast formation [16]. To sum up, these findings from in vitro and preclinical models suggest a potential role of FSH, independent of the well-documented role of estrogen in the regulation of BMD.

Epidemiological cohort studies [19,39] demonstrated a potential role of FSH in bone health, and interestingly, it appears that FSH compared to other sex hormones is the strongest mediator and predictor of bone loss during the menopause transition. The SWAN study [17] demonstrated that bone loss during the perimenopause can be predicted by changes in serum FSH levels, but not other reproductive hormone levels including E_2_. Furthermore, in perimenopausal and early postmenopausal women, BMD and bone turnover markers were inversely correlated with serum FSH levels but not serum E_2_ [39]. Discordant with the previous finding, the present study showed that spine and hip BMD were statistically significantly correlated with both FSH and E_2_ across all menopausal groups, albeit weakly (FSH: spine r = −0.341; hip r = −0.271; and E_2_: spine r = 0.274; hip r = 0.256, all *p* < 0.001). However, when we ran correlations only in perimenopause and early postmenopausal groups, correlations between BMD and FSH appeared to be stronger, while E_2_ lost its significance (Data not shown). More intervention studies are necessary to investigate the direct, mechanistic role of FSH versus E_2_ on bone metabolism in women.

### 4.3. Potential Limitations

The present study has limitations to be considered. The first important limitation was that this is a secondary analysis of baseline DXA data from a study on the biological mechanisms underlying vascular dysfunction with estrogen deficiency and aging in healthy women [20]. The original study was not powered to determine differences in BMD in spine and hip sites nor to test the associations of BMD with FSH or other sex hormones. Thus, the findings of this secondary analysis should be interpreted cautiously. Second, the present study used a cross-sectional study design, which precludes discussion of causality. The purpose of the present analysis was to assess BMD in different stages of the menopause transition, and to determine the correlations with sex hormones, specifically FSH. The menopause transition is related to changes in cardiometabolic and other factors that could affect BMD, yet it was beyond the scope of this analysis to assess these variables. Third, mass spectrometry may have provided more precise measurements of E_2_, particularly in peri- and postmenopausal women. Of the 141 women included in this analysis, 28 had E_2_ levels below the lower limit of detection (i.e., 10 pg/mL). When this occurred, a value of 10 pg/mL was used. However, the immunoassay used in the present study may be sufficient for measuring E_2_ levels in pre- and perimenopausal women, which comprised the majority of our cohort, and average E_2_ levels were lower across menopause stages, as expected. Finally, despite statistically significant Pearson and partial correlations between BMD and FSH and E_2_, the values were weak, and thus, the findings should be interpreted cautiously.

## 5. Conclusions

This study confirms the menopausal transition results in bone loss of the spine and hip. In addition to estrogen, we also demonstrated that FSH was significantly correlated with BMD at both the spine and hip in all menopausal stages. Potential mechanisms underlying menopause-related BMD loss such as FSH should be explored in human experimental studies.

## Figures and Tables

**Table 1:** Subject characteristics.

Variable	Pre *n* = 30	EPeri *n* = 31	LPeri *n* = 30	EPost *n* = 24	LPost *n* = 26	*p*-Value
Age, yr	38 ± 6	50 ± 3	50 ± 4	55 ± 3	62 ± 4	<0.001
Weight, kg	66.3 ± 10.0	71.4 ± 11.0	67.4 ± 11.9	71.9 ± 13.4	66.8 ± 13.4	0.25
Height, cm	163 ± 6	165 ± 6	166 ± 8	164 ± 6	161 ± 8	0.10
BMI, kg/m^2^	24.7 ± 3.7	26.1 ± 3.9	24.5 ± 3.9	26.7 ± 5.2	25.6 ± 4.6	0.27
Lean mass, kg	42.2 ± 3.6	44.3 ± 5.7	40.5 ± 5.1	42.2 ± 6.5	38.8 ± 5.5	<0.01
Fat mass, kg	21.6 ± 7.7	24.7 ± 6.9	24.8 ± 8.1	27.5 ± 8.2	25.3 ± 9.6	0.12
BMC, kg	2.14 ± 0.27	2.33 ± 0.34	2.08 ± 0.26	2.06 ± 0.22	1.92 ± 0.37	<0.001
Estradiol, pg/mL *^,†^	79 (64–104)	70 (37–141)	34 (10–94)	11 (10–14)	10 (10–14)	<0.001
Estrone, ng/dL *^,†^	61 (41–70)	60 (34–88)	43 (30–69)	25 (23–32)	26 (23–37)	<0.001
FSH, μIU/mL ^†^	6.5 ± 3.4	22.0 ± 30.0	64.1 ± 35.5	73.4 ± 27.1	84.1 ± 33.3	<0.001
Progesterone, ng/dL *^,†^	0.4 (0.2–0.6)	0.5 (0.2–0.8)	0.3 (0.3–0.5)	0.3 (0.1–0.4)	0.2 (0.1–0.4)	<0.01
Testosterone, ng/dL *^,†^	24 (17–32)	22 (17–35)	20 (17–25)	17 (17–22)	17 (17–35)	0.22
Energy intake, kcal/kg ^‡^	25 ± 10	28 ± 8	28 ± 7	26 ± 7	27 ± 7	0.79
LTPA, MET h/wk *^,§^	8(4–18)	14 (8–18)	7 (5–14)	10 (5–14)	10 (6–25)	0.59
IL6, pg/mL ^‡^	1.28 ± 1.24	1.03 ± 0.65	1.10 ± 0.74	1.13 ± 0.87	0.86 ± 0.26	0.72
Brachial FMD, % ^†^	11.0 ± 3.6	8.4 ± 2.8	6.9 ± 2.1	6.1 ± 2.0	4.9 ± 1.8	<0.001

Data are mean ± standard deviation unless otherwise stated. * Data are median (interquartile range). Pre = premenopausal; EPeri = early perimenopausal; LPeri = late perimenopausal; EPost = early postmenopausal; LPost = late postmenopausal; BMI = body mass index; BMC = bone mineral content; FSH = follicle stimulating hormone; LTPA = leisure time physical activity; MET = metabolic equivalent; FMD = flow-mediated dilation. ^†^ Sample sizes of 117, ^‡^ Sample sizes of 85, ^§^ Sample sizes of 115.

**Table 2:** Spine and hip bone analysis.

Variable	Pre *n* = 30	EPeri *n* = 31	LPeri *n* = 30	EPost *n* = 24	LPost *n* = 26	*p*-Value
**Spine**						
L1-4 BMD, g/cm^2^	1.014 ± 0.126	1.093 ± 0.146	0.971 ± 0.103 ^b^	0.949 ± 0.132 ^b^	0.918 ± 0.167 ^b^	<0.001
L1-4 T-score	−0.3 ± 1.1	0.4 ± 1.3	−0.7 ± 0.9 ^b^	−0.9 ± 1.2 ^b^	−1.2 ± 1.5 ^b^	<0.001
**Hip**						
Neck BMD, g/cm^2^	0.799 ± 0.106	0.835 ± 0.119	0.767 ± 0.089	0.727 ± 0.099 ^b^	0.684 ± 0.097 ^a,b,c^	<0.001
Troch BMD, g/cm^2^	0.699 ± 0.096	0.719 ± 0.105	0.670 ± 0.074	0.661 ± 0.096 ^b^	0.617 ± 0.091 ^a,b^	<0.01
Total BMD, g/cm^2^	0.932 ± 0.119	0.970 ± 0.124	0.899 ± 0.106	0.875 ± 0.126 ^b^	0.821 ± 0.122 ^a,b^	<0.001
Total Hip T-score	−0.1 ± 1.0	0.2 ± 1.0	−0.4 ± 0.9	−0.5 ± 1.0 ^b^	−1.0 ± 1.0 ^a,b^	<0.001
**Prevalence of LBM and Osteoporosis**						
LBM, n (%)	11 (36.6)	9 (29.0)	15 (50.0)	13 (54.2)	14 (53.8)	
Osteoporosis, n (%)	0 (0)	0 (0)	1 (3.3)	3 (12.5)	7 (26.9)	

Data are mean ± standard deviation (SD) unless otherwise stated. Pre = premenopausal; EPeri = early perimenopausal; LPeri = late perimenopausal; EPost = early postmenopausal; LPost = late postmenopausal; L1-4 = lumbar 1-4; BMD = bone mineral density; T-score = (patient’s BMD-mean BMD of ~30 year-old healthy population)/SD of ~30 year-old healthy population. LBM = low bone mass, T-score < −1.0 and > −2.5; Osteoporosis = T-score ≤ −2.5; ^a^ *p* < 0.05 versus Pre; ^b^ *p* < 0.05 versus EPeri; ^c^ *p* < 0.05 versus LPeri.

**Table 3:** Correlations of bone mineral density with age, hormones, energy intake, and physical activity among all groups.

	Spine BMD	Total Hip BMD
**Pearson Correlations**		
Age	−0.147 ^†^	−0.242 **
Estradiol	0.274 **	0.256 **
Estrone	0.239 *	0.227 *
FSH	−0.341 ***	−0.271 **
Progesterone	0.155 ^†^	0.173 ^†^
Testosterone	0.072	0.076
Energy intake	−0.015	−0.063
LTPA	−0.008	0.050
**Partial Correlations of BMD with FSH and Estradiol**		
FSH vs. BMD	−0.341 ***	−0.271 **
Adj for Age	−0.348 ***	−0.174 ^†^
Adj for Estradiol	−0.228 *	−0.148
Adj for Estrone	−0.270 **	−0.195 *
Adj for Progesterone	−0.320 ***	−0.244 **
Adj for Testosterone	−0.337 ***	−0.265 **
Adj for energy intake	−0.366 **	−0.271 *
Adj for LTPA	−0.359 ***	−0.307 ***
Estradiol vs. BMD	0.274 **	0.256 **
Adj for Age	0.261 **	0.161 ^†^
Adj for FSH	0.086	0.118
Adj for Estrone	0.141	0.127
Adj for Progesterone	0.237 *	0.212 *
Adj for Testosterone	0.265 **	0.246 **
Adj for energy intake	0.249 *	0.248 *
Adj for LTPA	0.279 **	0.259 **

BMD = bone mineral density; FSH = follicle stimulating hormone; LTPA = leisure time physical activity. ^†^ *p* ≤ 0.10; * *p* < 0.05; ** *p* ≤ 0.01; *** *p* ≤ 0.001.

## Data Availability

All the data presented in this study are available on request. The data are not publicly available due to patient’s privacy.
